# Population Preparedness for Disasters and Extreme Weather Events as a Predictor of Building a Resilient Society: The Slovak Republic

**DOI:** 10.3390/ijerph18052311

**Published:** 2021-02-26

**Authors:** Michal Titko, Jozef Ristvej, Zenon Zamiar

**Affiliations:** 1 Department of Crisis Management, University of Žilina, 010 26 Žilina, Slovakia; jozef.ristvej@fbi.uniza.sk; 2 International University of Logistics and Transport, 51-168 Wroclaw, Poland; zzamiar@msl.com.pl

**Keywords:** disasters, preparedness, public health, risk perception, disaster awareness, resilience building, questionnaire survey

## Abstract

The current increase and severity of the natural disasters whose effects on the public health are likely to be even more extreme and complex, requires enhancing and developing the disaster preparedness on the population level. In order to be able to do so, it is inevitable and determinative to know the factors that affect people’s preparedness on the population level. Therefore, the objective of this article is to present the results from assessing the factors related to the population preparedness for the disasters on a sample of citizens living from the Slovak Republic. Our research is based on the exploration of the questionnaire survey’ results aimed at investigating the preparedness and preventive proactive behaviour of the population against the disasters. The search for the initiators of such a behaviour and assessment of the influence of various aspects (e.g., the respondents’ experience with disasters, their vulnerability to disasters, the risk awareness, the perception of the disaster risks in the changing environment, etc.) on the respondents’ behaviour against disasters is the main part of the article and is supported by the statistical analysis. The results of the survey suggest that the disaster risk awareness and overall disaster preparedness level is rather poor and the population is inactive. The proactive behaviour of the respondents against the disasters is partially affected by some of their personality and socio-economic characteristics, especially the younger respondents currently incline more to adopting the protective measures. In addition, other aspects, e.g., the negative experience with the disasters in the past influence the preparedness. However, the impacts must have been relatively serious for the proactive behaviour to be influenced. The influences of other aspects as well as the possible methods for improving the disaster preparedness and the possibilities of increasing the resilience of the population as a whole are also discussed in this article.

## 1. Introduction

The recent research results on extreme weather events, disasters, and climate change effects show that the crisis events and disasters will increase in frequency as well as in magnitude [1,2,3,4,5,6,7,8,9,10]. Central Europe, including the Slovak Republic (SR), is one of the exposed and vulnerable regions to the impacts of the hazards related to climate change [10,11]. In the country’s history, there are several examples of major disasters; mostly floods (e.g., in 2006, 2010, and 2013) or storms/whirlwinds (2004, 2017, and 2019) [12]. According to the statistics by the Ministry of Interior in the SR, there were 1560 natural disasters from 2013 to 2019, of which more than 70% were floods [13]. The number of the disasters during the time period followed was fluctuating with a moderately increasing tendency [13]. In the SR, the changing nature of the climate has been transparent since 2013 [13]. In connection with the climate change: (1) the nature of floods is changing—local flash floods are more common (it causes ever greater problems for the rescue services to provide assistance to the population) and (2) the influences of the weather are more visible (extreme temperatures, heatwaves, drought, and related forest fires have been reported in higher numbers) [14]. The evidence and the prognoses of the future occurrence and possible disaster consequences show that floods are the major threat for Slovakia, and therefore, this study aims especially at this phenomenon.

As a reaction to the development of the climate change, several frameworks of disaster risk reduction (DRR) (e.g., EU strategy (2013), Sendai Framework (2015), Paris Climate Agreement (2016), or the Agenda 2030 Sustainable Development Goals) have been adopted by the international organisations [15,16,17,18,19]. One of their supporting pillars is the building of the resilient society against future disasters [15,16,17,18,19]. Such policies or frameworks should enable the public (the society and individuals) to become more resilient against [20,21] or less vulnerable to the impacts of disasters [21,22,23,24,25]. The society-engaging in the DRR initiatives have been shown to help achieving that purpose [26,27]. Therefore, much attention (from governmental authorities and academic community) is paid to the understanding of the people’s role in the DRR system and to the possibilities for strengthening their position (e.g., in terms of engagement or preparedness) in this system [28,29,30,31,32,33,34,35,36].

The abovementioned international initiatives were followed and “cast” to national policies and activities for improving the level of the society protection against climate change influences [37]. In the SR, the society-oriented initiatives of building DRR or disaster vulnerability reduction are not well recognised yet [38,39]. So far, the Ministry of Interior (as the DRR guarantor) almost does not deal with the issue of the climate change and its impacts [39]. The Strategy of the Slovak Republic for Adapting to the Climate Change from 2018 is an exception [40]; however, it was submitted by the Ministry of Environment. The document contains a part dealing with the risk management and disaster management. The section about DRR did not occur in the first issue from 2014. The goals that can be found in this part are general, especially if we speak about the area of the disaster resilience and population’s preparedness. There are only intentions about (1) building of a knowledge base, based on the improved public awareness and education about the risks and ways how to minimise their consequences; (2) increasing the level of cooperation between the individuals, communities, and organizations; (3) support for the scientific research and the implementation of the research results. For the time being, there is no plan for realising these intentions of the strategy.

In the SR, there is lack of effective public participation in developing resilience to disasters related to the climate change [39]. During the last decade, the SR is primarily oriented on improving the professional rescue system (also priority of the aforementioned strategy) at the expense of the conceptual and strategic development of the DRR (there was no updated conception until 2015) and preparedness of the whole society [39]. However, the forces and resources of the professional rescue system will be probably insufficient (as shown also by the examples from the SR), especially in such cases that exceed the common scope (large-scale disasters, e.g., climate change scenarios) [41,42,43]. This system can fail in providing the life and health protection of the citizens and in ensuring the population’s needs in the full extent and adequate time [41,42,43]. The people’s preparation for such a situation is therefore inevitable [27]. Thus, disaster preparedness of the population is assumed as one of the important pillars of the community-based and society-engaging approaches to the DRR and building the resilient society for future disasters [44,45].

This research is aimed at the adults. No systematic approach or strategic educational framework, increasing disaster awareness or resilience against disasters’ consequences is determined for the adult population. As a whole, the tasks connected with improving the population preparedness for disasters are carried out only in compliance with the duties resulting from the legal regulations and other directives. There are no initiatives, policies, or strategies in the area of improving the resilience of the society against disasters and no initiatives that would lead to any strategic development of the populations’ preparedness in this area. The preparation of youth (university students) and adults for the disaster protection is carried out (in limited extent only) by the district offices, legal entities, and natural persons—entrepreneurs, self-governing regions, municipalities, and humanitarian associations (Slovak Red Cross, Office of Civil Protection of the SR) [46]. The municipality plays an important role in the DRR, as it is the primary point of contact for its population in terms of subsidiarity and possibility to build resilience. The content and form of the population preparedness for disasters and emergencies is chosen by the municipality itself [47]. These are usually training sessions, discussions, workshops, meetings, seminars, competitions of young rescuers, demonstrations of rescue techniques, and rescue activities, as well as local TV programmes. The population living in the most vulnerable areas of the SR are involved in the preparation activities in the framework of the possible realisation of the Population Protection Plan that is worked out by the municipality in collaboration with the competent bodies. The Population Protection Plan contains tasks, measures, and procedures to ensure the protection of the population during a disaster [46]. This plan represents suitable means for building the society’s resilience and implementing particular policies for strengthening this idea, but currently, the population’s involvement into this plan is minimal and optional, and it is trained only sporadically. The process of preparation and education in this area is not continuous and flexible, and therefore, it cannot reflect the changing environment and current development. The Slovak government neither investigates nor assesses the level of the population disaster preparedness or the risk awareness of the population (based on the personal communication with the representatives of the Ministry of the Interior and local state administration and self-government bodies) [39].

In Slovakia and in the immediate neighbourhood (Poland, Hungary, the Czech Republic, the Ukraine, Austria), despite the aforementioned threat of the increased flood frequency due to the climate change [10,11,13], the public preparedness and resilience factors remain unexplored to a large extent. The studies by Bubeck et al. [48] and Kellens et al. [49] (more than 70 studies) include only one dealing with the relation between risk perception and insurance in Poland [50]. At the same time, there are no studies in the region examining the differences of the risk perceptions related to the climate change. Given the lack of investigation in that region, and the mentioned shortcomings of the DRR system in Slovakia, we conducted a questionnaire research with the goal to achieve a picture about the population preparedness for disasters and what it is affected by. The work aims at: (1) providing basic understanding of how the individuals are aware of the flood risk in Slovakia, how they perceive the flood risk, and whether they are preparing for the future floods; (2) exploring the correlations of the demographics, the previous flood experiences, risk awareness, and risk perception with respondents’ protective behaviour (through selected variables, see Table 1) in order to find the predictors of this behaviour and potential for enhancing the resilience of the society; (3) exploring the differences of the risk perceptions related to the climate change (a comparison of young generation and other adults), especially whether young people feel to be more threatened by the climate change and currently to be more aware of the climate change risks; and (4) exploring the interest in the disaster protection area.

The summary of the findings can be a basic point for designing particular initiatives that can systematically support the DRR activities in the SR and enhance disaster preparedness of the population.

## 2. Disaster Preparedness-Theoretical Framework

There are several definitions of the population’s (individuals’) preparedness for disasters. One of them is the definition according to the Slovak law that should provide enough space for a comprehensive realisation of the activities connected with preparing the population and, at the same time, to require their fulfilment, however, the second requirement is not fulfilled [46,47]. In the SR, the preparedness of the population for disasters and extreme weather events is understood to be the preparedness of the population for self-protection and mutual assistance, as well as preparedness for administering the first aid [46]. Specifically, it is a “purposeful and continuous process of preventive, educational and promotional activities, and theoretical and practical training” [47]. These activities are intended to enable the population to acquire the necessary theoretical knowledge, practical skills, and habits of self-protection, in other words, “assistance with their own means and forces that aims to protect the persons themselves and their immediate surroundings and to mitigate or prevent the effects of a crisis event” [46], “but also assistance to others in need” [47].

This definition shows that the theoretical knowledge, practical skills, and capabilities of the self-defence and protection of the close persons in the case of disasters are important aspects of the preparedness. According to Kitagawa, the preparedness can be understood as the knowledge and capacities developed by the subjects (individuals and organisations) to effectively anticipate, respond to and recover from the impacts of disasters [51]. The common denominators are thus the knowledge and capacities (including the skills). It is undisputable that the knowledge of certain factors, activities and processes can help improve the prevention and also the reaction and activities connected with recovery after the crisis event [52]. A close linkage or subordination with the aspect “knowledge” can be seen also in the case of other authors primarily with the aspects of the risk or disaster “perception” [53,54,55,56,57,58] and “awareness” [59,60,61]. The knowledge forms the risk perception and the knowledge is shaped by the perception, and similarly it is in relation with the risk awareness. Already Cutter (1993) (cited in Murphy et al. (2005)) defined the human perception of the risk as a process that links individual judgements of the degree of danger with an action [62] (what people think about the risks or disasters). An assumption of the disaster occurrence (often observed as the possibility or likelihood of the disaster occurrence, e.g., [63]) or fears of the future disasters may lead into activities to increase the level of our own safety. Another part of disaster perception can be seen in a person’s belief in his or her own ability to achieve the results/goals-in our case, the ability to overcome a crisis successfully (known also as self-efficacy) [64,65]. The combination of these factors could influence the choice of the cognitive patterns that people use in such situations.

The research of the disaster awareness (what people know and understand about disasters) can reveal an insufficient or erroneous perception of the links people have in the area or can contribute to clarifying the lack of knowledge (hazard-prone areas awareness, protection options awareness, disaster solution-procedures awareness, warning signals awareness, etc.) [66,67,68,69]. The detected facts are also a subject of our research. Besides the wide investigation of the disaster risk perception and disaster awareness, the research is often bound to the local conditions (vulnerable or threatened areas and vulnerable or more sensitive groups of population) [70,71,72].

The aforementioned aspects often depend on the previous experience of people with disasters [73,74,75,76,77], although it is not always the case. Already, Weinstein (1988) [78] and also other authors (e.g., [57,73,79,80]) described how the experience affects the disaster risk perception and awareness. The previous experience can also affect people’s behaviour (what people do after the disaster experience). There is evidence showing the fact that the experience with disasters can motivate people to the mitigation behaviour (defined by the UNISDR [81]) in form of not only reactive but also proactive behaviour, e.g., realising measures for coping with disasters, taking precautionary action to withstand the disaster effects, or creating reserves for survival [73,80,82,83,84]. However, in some cases, it can be the “experience-adjustment paradox” [84]. The situation whose intensity and destructive force was low can lead to the “incorrect” experience and an erroneous interpretation (or expectations) of the links in the future [84]. From this perspective, it is important to study the severity of a disaster experienced in order to address possible misleading in the individuals’ interpretations. The experiences can be a stimulus for a certain type of behaviour, and in this way, they affect the perception [61], awareness, knowledge, and preparedness of the individuals or population as a whole [85]. The previous personal experience as a behavioural predictor or a factor influencing a whole range of other aspects (risk awareness and perception) connected with disaster preparedness must be investigated thoroughly [84,86,87] because the aforementioned statements are not confirmed by all studiers and can be different according to the type of investigating the initiation event.

From the behavioural point of view, the preparedness of the population is analysed on the basis of the socio-cognitive model [88]. It deals with the fact that the preparedness represents a result of the three-phase justification process [88], i.e., the motivation to prepare, forming intentions to prepare, and their conversion into the actual preparation. This approach (or a similar one) still involves a certain amount of distortion because the behavioural assessment of a certain group of respondents need not be applicable for other cases [89]. It can be caused by the fact that there are links with other attributes of the personality itself and also the surroundings at the given moment of making decisions. On the one hand, the respondent had other intentions, but on the other hand, the real situation will change his/her probable behaviour (decision/behaviour change). The studies, e.g., [70,73], investigate more the reactive behaviour than the proactive behaviour, and most frequently, it is realised on the hypothetical level (What would you do for the earthquake not to happen?). It enables mainly the comparison of the assumed behaviour with the recommendations of the competent authorities or stated strategies, however, it can face the aforementioned “decision/behaviour change” problem. Our study was aimed especially at the proactive behaviour (particular measures that the respondents carried out in the past or are realising continuously).

The other principal pillar of the preparedness is the aspect of the “capacities” that can be identified with the views of other authors under different names (e.g., objective preparedness [90]) and partial different from the content point of view (inventories, material resources [91], financial reserves [92], evacuation capacities [57], etc.). Considerable differences can be found in the understanding of the capacities from individuals’/household point of view (interest of this study) or in understanding from the point of view of the rescue and recovery organisations, municipality, or the state. The second view takes into account (besides the material) also the available personal capacities for solving situation or emergency. Russel et al. [91] define in their study (on an example of the earthquake) three aspects of the preparedness—the structural, planning, and survival one. All of these three aspects refer to the aforementioned proactive behaviour because this behaviour can result in taking measures before the situation itself: (1) by hard (structural) intervention measures (the structural aspect, e.g., protection constructions or surface adaptations, structural elements, and technologies [81,93,94]) and (2) by soft (non-structural) intervention measures (the planning aspects, e.g., plans, instructions, policies, and methods [57,81]). (3) The creation of the reserves is similarly part of the preparedness—the capacities (survival aspect). The reserves should serve both the individuals and households for withstanding the disaster effects, and the common sources of food and water, but also information (radio), are missing. The replacement sources of energy can be also taken into account (batteries and aggregates), certain tools, various means or equipment that would serve for solving the situation (water pumps, lamps, and packs with sand). From the point of view of the evacuation capacities, Niwa et al. [61] include also the physical capability to evacuate or to help the family members, friends, community members, or local government and the professional rescue services with evacuation into the capacities. These individual capabilities are important in those cases when the affected area and community is dependent on itself. However, in those cases when the evacuation is necessary, the material commodities will be necessary only for a short time, and after this, they seem to be low effective. Due to this fact, the recovery and return to the given area can be demanding and the capacities the returned people will need are especially the financial reserves [95], e.g., the insurance payment or common financial reserves [96,97,98]. The financial reserves are not important only due to the possible consequences of disasters but also in the case of any unplanned expenses of the individual or family. The creation of not only these reserves but also other types of the reserves or realising various structural or non-structural measures depends to a considerable extent on the individual’s or family’s economic possibilities [87,99] and also on perceiving the possibilities of occurring the negatively performing situations in the people’s life or the attitude to the risk of such events, i.e., we are returning back to the perception and awareness. The interdependence between these aspects can be assumed. The majority of the previous studies investigate the economic aspect in terms of the income. In our opinion, a more suitable way is to compare whether people are able to recover from the different levels of the disaster impact. This is dictated by a practical consideration that after the disaster, those people, who are economically stable, should be able to return to previous state of their daily life—everyone has a different life standard and therefore everyone needs a different amount of money for that level.

According to the aforementioned facts, the disaster preparedness refers to the preparation of activities and resources designed to improve the system capacities and protection abilities in advance, which corresponds to the interpretation of the resilience as the ability of a system to resist disruption by reducing the initial negative impacts (absorbability), adapting itself to disruption (adaptability), and recovering from disruption (restorability) [100]. The relation between preparedness and resilience can be seen by the implication of the resilience as the ratio of this preparedness to vulnerability of the system under consideration [100,101,102]. The higher the preparedness level, the higher the resilience and the higher the vulnerability, the lower the resilience [102]. Thus, the discussed preparedness aspects (risk perception, awareness, disaster experience, as well as different forms of the capacities) could contribute to enhancing the disaster resilience [52,102] and are of the main interest of the survey conducted.

On the whole, within the previous studies, the same or very similar aspects (perceptions of risk, awareness of risk, experience, and demographic) are used to examine the disaster preparedness. The studies apply similar conclusions, but quite often, these studies address only some aspects of preparedness separately or a partial combination thereof. The common aspect is the socio-demographic aspect. The present study examines the impact of all the aspects on the protective behaviour. In the literature, the given aspects are mostly expressed by several variables. They differ more or less in the content or the manner of expression. Therefore, after analysing the suitability of specific variables, we have modified some variables (e.g., “economic situation” and “protective behaviour”) in order to achieve the objectives of the work and, at the same time, to verify the suitability of such modified variables. There are also contradictory results within the literature, which prevent us from applying the results for our region without conducting the research for this region.

## 3. Methods

### 3.1. Research Methods and Questionnaire Survey

The authors created a structured, self-administered, and anonymous questionnaire with the goal to identify and investigate the predictors (determinants) of people’s disaster preparedness and to assess the importance of these predictors for the decision-making process of individual persons. The research was realised from January 2019 to July 2019. Due to the absence of a comprehensive sampling frame, the questionnaire was distributed by internet through a direct link to the questionnaire in two forms: (1) via university student accounts (randomly selected students) and (2) a list-based sampling: a link was distributed to randomly selected e-mail addresses from the e-mail list (non-student list; permission of the potential respondents was required). The non-student email list was obtained by the snowball technique (the faculty students were asked to contact acquaintances (18+) with a request to provide their e-mail address for the purpose of the questionnaire distribution). The questionnaire was approved by management of the faculty and the questionnaire answers were not paired with the e-mail addresses so the anonymity of the respondents was ensured. Provider of the survey service secured the data in a server. Sample biases were related with this kind of sampling but this effect was minimalised because this study is focused on investigating the relationships between variables using the samples from the SR rather than on investigating the preparedness level for people of the SR. A sample size of 794 was calculated for a 95% confidence interval and 3.5% error of margin. Due to different questions in the research framework, the number of respondents (n) can be different because some questions were not compulsory and others were not answered because of individual reasons.

Compared with other studies, this questionnaire was created in a more complex way because it was aimed at various hazards (floods, whirlwinds and strong storms, snow calamities, heatwaves, drought, earthquakes, landslides, and forest fires). However, this study investigated only the understanding of the respondents in the area of the floods as the most common disaster type in the SR [13]. We are considering comparing the differences between the disaster types in further research.

In the first part, the questionnaire reflected the respondents’ basic personal characteristics. These characteristics were chosen by analyses of other studies. This study used information about the gender, age, education level, social status (youth and adults), location of living (urban and rural), type of living (flat and house), number of disabled people or people requiring higher care in household, and the household’s economic situation (it was not measured by income but through the ability to recover from different crisis situations). As it was stated in the introduction, the focus of the study was on the adults. In our case, the youth were the subcategory of the adults. This group was for the purpose of this research divided into two groups: the youth and the adults (variable social status, see Table 2). The youth represented the students at the universities more than 18 years old. It was important to us to separate this group from two reasons. (1) In the SR, the students from the basic to high school received basic information about the disaster protection during their study but not at the universities. However, the university students were assumed as the population group, which can have still good information about the disaster protection and risk reduction in comparison to the other adults because of their recent education. (2) It was assumed that the university students lack disaster experience due to the age (in comparison with other adults). For these reasons, the study separated the survey participants within adults into these two social groups.

The next parts of the questionnaire addressed the aspects and variables of the preparedness and proactive behaviour selected (Section 3.2) on basis of the literature review (Section 2) and research objectives.

### 3.2. Key Aspects and Variables of the Study

Based on the analysis of the aspects affecting the population preparedness for disasters (Section 2), we investigated the following aspects: (a) the experience with disasters and extreme weather events, (b) disaster awareness, (c) the risk perception, and (d) the proactive behaviour and adoption of the protective measures. At the same time, the questionnaire reflected some elements of the society vulnerability because we assume certain links with the level of the population preparedness. The particular main variables connected with the defined aspects are shown in Table 1.

The survey also involved a section that did not deal directly with population preparedness. It was aimed at other aspects that were connected rather with investigating the background of the interest/no interest in this area and reasons of the interest/no interest. The facts about the most preferred methods of acquiring information about disasters and the protection possibilities were collected as well. Subsequently, the most accessible and preferable variants of strengthening the preparedness and education of the population through those methods could be identified.

### 3.3. Statistical Analysis

The correlation analyses and regression analyses were conducted. Using the Pearson’s (r) and Spearman’s (r_s_) correlations, we investigated the relations between the dependent variable (protection action adopted) and further ordinal or interval variables through the bivariate analysis. The respondents answered the questions concerning the protection action adopted as follows: “yes, I adopted some measures” (they could be the already mentioned structural measures, non-structural measures, or creation of capacities) or—“no, I adopted no measures.” For the purposes of investigating the dependences (the Pearson’s and Spearman’s correlations), the dependent variable was altered into the numerical value “1” for the “yes” answers and “0” for the “no” answers. To describe the strength of the correlation, the guide that Evans (1996) suggests was used [103]. The relation between the nominal variables was investigated using the Chi-square test and calculating the association coefficient (V—Cramer’s measure of association).

This study looked for and investigated the predictors (causes) of the proactive (protection) behaviour with the goal to assess the importance of these factors for the decision-making process of individual persons. In order to investigate these predictors, the multiple regression analysis was performed. As the dependent variable was dichotomous, the logistic regression analysis was more appropriate. Regression analyses were conducted for the basic demographic characteristics and for each of the preparedness aspect separately.

## 4. Results

First, the results of descriptive statistics are presented for various aspects and variables, followed by the results of examining the relationships between the dependent variable “the preventive and mitigation measures taken” and explanatory variables.

### 4.1. Descriptive Statistics

#### 4.1.1. General Attributes of Respondents

Our study investigates first, the basic demographic and socio-economic characteristics of the respondents including the variables from Table 2. These variables can have significant influence on the protective behaviour and show differences between groups of the population concerning the disaster preparedness. The majority of participants were male (52.39%). The mean age was 29.35 (SD = 11.36) years, which was affected by the focus on the university students (aged mostly from 18 to 24 years; they create 52% of the sample). Almost 40% of the participants had already an academic degree. About 45% of respondents are university students. There is approximately the same proportion of people living in the urban and rural area. More than 57% of the respondents live in a house. On average, respondents are living in the households with one (0.63; SD = 0.96) disabled family member or with a person with increased need of care; however, most often without such a person. Economic situation of the respondents is rather below average (2.25 from 5), which suggests rather low financial reserves for recovery after a disaster. The sample’s characteristics differ from the actual characteristics of the Slovakia’ population, mainly due to the higher proportion of the university students, but it was the aim of the authors to investigate these differences.

#### 4.1.2. Previous Disaster and Extreme Weather Events Experience

The research participants show a relatively high share of the direct experience with floods. Over a half of the respondents experienced the floods (57.05%). Out of those respondents who experienced a flood, even 58.28% of them experienced it several times.

The “preparedness adjustment paradox” indicates that not any type of the experience can affect the increased motivation for a proactive behaviour and realising the preventive measures. Therefore, we were looking also for the answers to the question “Which worst impacts the respondents suffered if they had had experience with a disaster”. It serves us to learn how bad experience they really had. The scale from Table 1 for assessing the seriousness of the experienced impacts was used.

If the respondents had stated that they had experienced some flood impacts, the highest share of the worst impacts were “major damages” (48.48%). “The minor damages” have a share of 23.48% and then, “threats for life and health” (15.43%) and “the logistic complications” with 12.61%. It can be said that almost two-thirds of those who experienced some flood impacts had experienced an event with serious impacts on their property, surroundings, health, and life. Based on these facts, it can be said that floods are a very serious threat for the population safety, and it is an important issue to deal within the framework of strengthening the population’s resilience. The results of the survey indicate a big difference between disaster impacts related to the disaster type. The comparison of these differences is not part of this article but our intention is to do so in our further research.

The second important aspect connecting the seriousness of the experienced event is the necessity of evacuation of a certain part of the population from the endangered territory to safety. The research shows that only 8.31% of the respondents were evacuated in the past that is a relatively low number from the point of view of serious floods experienced. From the official statistics about disasters (e.g., year 2018 [13]), only 2% of floods were connected with the evacuation. It is similar for other years and even lower number is reported but it is important to say that information about evacuation is not mandatory to report in official statics. Therefore, it is difficult to compare it with the results obtained.

#### 4.1.3. Disaster Awareness

In this section, we investigated the aspects concerning disaster awareness (flood related) as follows: (1) the knowledge that people live in (or close to) the endangered territory (flood-prone areas)—53.16% were aware of the fact they are living in a territory endangered at least by one threat; (2) the flood map awareness—26.45% are aware of the content of the flood risk maps for their region; (3) the evacuation plan awareness—the respondents’ knowledge about the plans and procedures of evacuation is low, and less than 8% are aware of the evacuation plan content and the followed activities; 32% of the respondents are not aware of the content and connected activities; and more than 66% do not know that any evacuation plans even exist for their region; (4) the warning signal awareness—only 23.17% of the respondents know the warning signals, one third of them admitted they were not able to give a correct answer; and (5) the awareness about possibilities of protection against floods—recently only 27.7% have come into contact with the topic of protecting against floods and are aware of the possibility of protection against their effects.

#### 4.1.4. Risk Perception

The risk perception was evaluated by a five-segment assessment. The research participants were asked “Where do you fit on the scale from 1 to 5?” (5 means better or higher). The overall score was calculated as an average of these five items (the mean = 3.05). Moreover, the opinion concerning the rate of demonstration of the climate change (how the climate change manifests itself) in the SR, Europe, and worldwide was detected in Table 3.

#### 4.1.5. Preventive and Mitigation Measures Adopted

The proactive behaviour was addressed by the question if the respondents took certain measures (preventive or mitigation ones) that would protect them against the impacts of disasters or which would help them overcome the impacts quicker. The dichotomous answers were formed-yes/no. Furthermore, 66.1% of the respondents took no preventive or mitigation measures and the rest (33.9%) prepared for possible disasters through various measures or creating capacities for coping with them and survival. The respondents were also asked about the particular method of preparation but as the answer was voluntary, only a few of them answered it (about 8%), there were answers that indicated preferably the material preparation and protection of the households (water pumps, alternative sources of energy, protection of the estates, insurance, etc.). Based on this low amount of information, it was not possible to make any conclusions regarding to the methods of preparation as indicated, e.g., by Rusell et al. [91].

People are paradoxically interested in the area. Here, 79.09% of the respondents are interested in other information concerning the possibilities of protecting against disasters. It is necessary to make use of the interest in the given area and to strengthen the educational basis of the population [104,105,106]. It can be subsequently transformed into building a resilient society against various types of risks and disasters.

### 4.2. Relation between the Preventive and Mitigation Measures Adopted and Other Variables of the Study

The following sections and tables (Table 4, Table 5, Table 6, and Table 7) bring the results of investigating the relations between the dependent variable and other explanatory variables. These results offer a view at the stimuli that arouse the protective activities in relation to a risk of developing disasters. The results are divided into four research areas—areas related to the aspects of preparedness. Each area contains a correlation analysis as well as a multiple logistic regression analysis.

#### 4.2.1. Relation between the Preventive and Mitigation Measures Adopted and Basic Attributes and Characteristics of the Respondents

In the framework of investigating the relation between the “preventive and mitigation measures adopted” and the basic attributes and characteristics of the respondents, we identified the dependences, and they were significant almost for all attributes except for the gender and the number of disabled people or people with need of increased care in household (Table 4). However, the strength of these dependences is different. The moderate correlation (r = − 0.26; r_s_ = − 0.34, *p* < 0.01) was between the age and the dependent variable. The dependence was negative, and this fact shows that younger people tend to take protective measures more than older ones. Another important finding was the fact that there is an association between the variable “social status” and the dependent variable. The Pearson’ correlation coefficient was stronger (r = − 0.48, *p* < 0.01) and V = 0.23, *p* < 0.01.

It can be argued that on the reliability level of *p* < 0.01, there is a relation between the “economic situation” and dependent variable with the low to moderate dependence rate (r = 0.27, r_s_ = 0.21).

The positive correlation was expected between the “number of the disabled people or people with the need of increased care in the household” and the dependent variable. We assumed that the presence of disabled people in the household increases the activity towards protecting such a household. It is clear that the families having problems with persons with, e.g., mobility problems try to prepare for the situations that are not a commonplace (e.g., transfer problem) but an assumption was not confirmed for disaster preparedness activities (r = −0.03, *p* > 0.05; r_s_ = −0.02, *p* > 0.05). The reason for that can be also related with the mean value, which is only 0.63. On contrary, the number of people in the household affects the preventive behaviour (r = 0.13, r_s_ = 0.15, *p* < 0.01).

After the correlation analysis, we investigated the influence of the explanatory variables on the dependent variable with a multiple logistic regression analysis. It identifies the factors extensively influencing the preventive behaviour (Table 5). For the same reason, the regression analysis was done also for other preparedness aspects of this study.

The results indicate that the most relevant factors of the basic attributes and characteristics of the respondents with influence on the preventive behaviour and disaster preparedness are the age, social status, and economic situation (Nagelkerke R^2^ = 0.33; Hosmer and Lemeshow test: Chi-square = 9.12, Significance = 0.33).

#### 4.2.2. Relation between the Preventive and Mitigation Measures Adopted and Disaster Experience Variables

Regarding to the already realised research it was desirable to find out how and if at all the different disaster experience would reflect in the behaviour or in the effort to prepare for further similar events that people had experienced before. The results are in Table 6. The flood-related results were used only. The results indicate that the fact if the respondents were evacuated or not in the past does not affect the dependent variable. Based on another aspect “number of events experienced” we can say on the reliability level of *p* < 0.01 that there is a relation between the variables investigated but the dependence rate is relatively weak (r = 0.12, r_s_ = 0.14). A stronger relation was calculated between the dependent variable and the worst experienced consequences of disasters. The Pearson’ correlation (r = 0.44, *p* < 0.01; r_s_ = 0.47, *p* < 0.01) indicates that the people’s experience is not enough for a person to begin to do something and to be active also within the society and to build its resilience, however, the negative side of this experience plays an important role in his/her behaviour in the future. We can assume that further negative events with serious consequences would arouse an active preparation for disasters.

Disaster experience variables were also examined for their effect on preventive behaviour by the regression analysis (Table 7). The results confirmed that the worst disaster experience had the most significant influence on the preventive behaviour and adopting the protective measures (Nagelkerke R^2^ = 0.26; Hosmer and Lemeshow test: Chi-square = 16.35, Significance = 0.03).

#### 4.2.3. Relation between the Preventive and Mitigation Measures Adopted and Disaster Awareness Variables

The awareness and knowledge of the population about the threats, flood-prone areas, warning signals, plans, and procedures of the evacuation and possibilities of protecting against floods are weak on the average. Table 8 clearly shows that the level of the relation importance between the dependent variable and the explanatory variables is significant for all of the combinations on the level *p* < 0.01. However, the association rates between the variables are low. The variables “flood map awareness” and “evacuation plan awareness” show a little bit higher rate of association. Several respondents say they have partial or complete knowledge about these properties and this fact could initiate their active behaviour. The higher flood maps and evacuation plans awareness can be primarily connected with the fact that the persons concerned had often direct experience with floods. The people need to experience something to be able to understand the importance of the theoretical knowledge. The given relation was confirmed in the previous part, and therefore, a partial duplicity of investigating these relations is possible.

The knowledge and awareness are not the driving engine of the changes in disaster preparedness (Nagelkerke R^2^ = 0.21; Hosmer and Lemeshow test: Chi-square = 8.12, Significance = 0.23) but the willingness to adopt precautionary measures is positively related in many cases with the risk awareness. The regression analysis also confirmed the significant influence of all the awareness variables (Table 9).

#### 4.2.4. Relation between the Preventive and Mitigation Measures Adopted and Risk Perception Variables

The majority of the investigated risk perception variables is not connected with taking protection measures by the population. (Table 10) The results of the correlation analysis prove that only “fears of occurring disasters in the future” and “subjective assessment of own preparedness for disasters” are the predictors of taking the protection measures. The strength of the dependence found is rather weak.

Regression analysis (Table 11; Nagelkerke R^2^ = 0.14; Hosmer and Lemeshow test: Chi-square = 8.01, Significance = 0.43) confirmed the significant influence on the preventive behaviour for previously identified variables (*p* < 0.01).

## 5. Discussion

The findings concerning the main aspects of preparedness and connected variables suggest that the population preparedness for disasters and extreme weather events is rather low. This complies with the subjective assessment of preparedness (mean = 2.60, SD = 1.01) and results of other studies (e.g., [70,73]). The results suggest that the disaster preparedness is based on the experience of people from previous events and on the knowledge and awareness about the risk rather than risk perception. However, the results will need further confirmation as this study was not oriented on all age groups of the population.

The youth (university students) introduced that they had adopted protective measures for protecting the households and relatives more than the adult group. This result is not fitting to a general understanding that older people tend to be more cautious about the risk [107]. Previous findings illustrate a positive correlation between the age and taking protective measures and adopting protective behaviour ([67,107,108]); although negative outcomes have also been found [109]. It was assumed that these contradictory findings can stem from the fact that the young generation feels to be more threatened by the climate change and is currently more aware of the climate change risks. However, this hypothesis is not supported by the results as the correlation between the age and the variable “rate of demonstration of climate change in the SR” is positive r = 0.15. Nevertheless, the additional research of the reasons for the interest in protecting against disasters showed that “the environment is changing very quickly and the development of the disasters is connected with the climate change” and “we can hear everywhere about disasters, so we should do something” are reasons that had a 41.37% representation in the answers and suggest relation of active behaviour with the climate change manifestation. Subsequently “the negative experiences from the past”, “the fear of our health, life, and property” as well as “the fear of life and life of our relatives” (33.18%) are important predictors of the interest in this area.

The results of this study are in agreement with finding that people with previous flood experience are more likely to adopt mitigation measures [80,110,111]. If we compare young people and adults in terms of the number of flood experience, there is no difference. The increasing number of floods in Slovakia in recent years [13] (especially flash floods) may have contributed to the number of experiences among young people and contributed to the active behaviour of young people as well. The young people start to be more aware of the necessity realising activities aimed at preparing for the future disasters. The disaster experience is a trigger for protective behaviour, regardless of age.

Shapira et al. [73] indicates that the population with a higher income have most probably an ability to engage themselves in their own protection against an undesirable event. This statement was confirmed in this study. However, this study used the variable “economic situation”, which do not refer directly to the income or a higher socio-economic status but on the ability to restore the household to its original state after impacts of a disaster. The people in a better economic situation have confirmed their intentions to ensure the household also before a disaster. The economic situation is not a factor that can be directly affected by the local or governmental authorities but the economically vulnerable groups of the population can be aimed by the specific measures to help them to be better prepared for disasters. The removal of the economic barriers and creation of a solidarity environment represent a potential for developing DRR and engagement of a wider part of the population. There is space mainly at the municipality level, which can work closely with such vulnerable groups but the resources at his level are very limited. Thus, a support from the local and the governmental level is still desirable.

The knowledge and awareness are important factors of disaster preparedness. The willingness to adopt precautionary measures is positively related in many cases with the risk awareness factors. It is consistent with some other studies [73,82,85]. The awareness remains an important element in enhancing disaster preparedness, however, the content of information provided and methods of information distribution are still questionable. Diakakis et al. (2018) recommend that awareness campaigns should stress the importance of floods through damage or fatality statistics, should illustrate the value of taking private protection measures for improving own safety and the resilience, and should target all citizens [70]. Knowledge of the imminent danger must be associated with sufficient information on how to deal with the situation, and at the same time, the individual must be able to act in the direction of this information. The limitations of such an activity should be removed by conceptual management and targeted help to the most vulnerable group of the population [24,73].

The majority of the investigated risk perception variables is not connected with taking protection measures by the population. The results of the correlation analysis prove that only “fears of occurring disasters in the future” and “subjective assessment of own preparedness for disasters” are the predictors of taking the protection measures. The findings are partly consistent (as only two investigated variables are detected as positively correlated) with several previous studies that claim that there is hardly any relation observed between the risk perception and realising the protection measures [48,110,112,113,114,115,116]. The findings are partly in compliance with the theoretical models (e.g., [82,117]). It is evident that stressing only the risk of flooding or the fact that people’s preparedness is low is not sufficient. People know about the possible risks in the future and that they are probably not enough prepared but it still does not change their behaviour. The fear has an effect on the protective behaviour but it mostly stems from previous experience what supports the statement that the disaster experience shapes the individual’s expectations of the future disasters and their behaviour [80]. Similarly, any flood experience has a significant influence on the rate of the self-assessment preparedness (r = 0.17, r_s_ = 0.17; *p* < 0.01). This assessment changes only minimally with the increasing number of experience, but in connection with the worst experienced consequences of disasters, this significant dependence rate is even stronger (*p* < 0.01, r = 0.26, r_s_ = 0.26).

If the population took any measures and, at the same time, they were assessing their preparedness level by a higher score, it could be assumed that they would feel in their municipality more safely, but the given assumption was not confirmed. It may be caused by the fact that the feeling of safety itself is disrupted by a stochastic nature of the threats [118] that can manifest itself in an unpredictable form and anytime.

Within perception of the climate change and its impacts in the SR, in Europe, and worldwide and the relation of this perception with the dependent variable, it is obvious that only the perception of the situation change in the SR leads to taking certain preventive measures or coping with them. People perceive threats of climate changes, but for them, it is relatively difficult to take any protective measures against impacts on the individual level, especially for the economically vulnerable population, and therefore, only weak relation between these factors was proved. The communication campaigns led by the governmental and local authorities will play an important role in enhancing future resilience to climate-related disasters and promoting appropriate, easy to implement, and affordable adaptation and mitigation measures. In this regard, this study does not investigate trust in the public authorities, but as the literature suggests [49,109], the population trusts in the institutions that developed efficient protection measures. People will later trust the information these institutions provide [49,109]. As it was mentioned in the introduction, in recent years, the efforts have been made to improve professional rescue services as well as flood protection measures in the SR, which have been well applied in practice [39]. This is a good starting point for building institutional trust and creating information campaigns for the stimulation of the public motivation and participation. However, the increasing number of disasters can disrupt the trust in the authorities as the measures made can be seen as insufficient by the public. Therefore, in our opinion, these activities (developing efficient measures, building trust, and disaster protection information base) should be realised simultaneously and continuously.

As it was mentioned above, there is an interest of the population in the disaster protection area. The question is how to satisfy/address it suitably. Through the additional questions in the questionnaire, it was found that people prefer more the indirect methods of acquiring information (80.96%), e.g., telephone applications (16.89%), the information material—mails/electronic form (12.60%), web sites (16.95%), leaflets, brochures, and other information materials—in the printed form (12.19%) and lectures by experts (22.33%). The direct methods (in-field trainings (7.79%) and the direct communication with the experts (11.24%)) created only the smaller segment. Due to the fact that the bad experience from the past events was the most significant predictor of the protective behaviour, to transfer this experience to the people who did not experience such a serious manifestation of a disaster will be very challenging. Just the direct methods seem to be the most suitable option. As the interest here is lower, promoting direct methods will be the crucial part of enhancing the disaster preparedness. The extension of practicing the Population Protection Plans (in terms of practicing frequency, extension of participants, tasks and related procedures, and potential disasters) could be an option, but it may be a too directive way for the public and may not meet with a positive response. In order to find a less directive way, the motivational and behavioural analysis should be made. The other methods (the indirect ones) have also their importance, especially to support the information base and disaster awareness.

### 5.1. Implications for Disaster Risk Management and Building of the Disaster Resilience

Given the results of the study, addressing the population awareness should be for the policy makers and the responsible authorities the basic point towards enhancing the disaster risk management and population preparedness. For that purpose, the all-aspect (from hazard-related to protection option awareness) approach should be preferred instead of selecting the most important factors as almost all awareness factors had a significant influence on the protective behaviour. Emphasising one awareness factor or only part of disaster awareness may fail in making the proper decisions due to limited knowledge of the issue. However, such an approach assumed the well-prepared and easy to access information base, information enlightenment as well as an appropriately selected information distribution method (some considerations can be found in the previous paragraph) for different groups of the adult population.

The subjective assessment of respondents’ preparedness was associated with the implementation of activities against the effects of floods. Motivating and especially supporting responsible preparation of individuals for disasters can obviously conclude in higher preparedness. It is of significant importance not only for individuals (they will feel safe and will be better prepared) but also for the state (more resilient population can conclude in saved costs), as the burden of disaster protection issue is currently on the shoulders of the state (from the governmental level to the municipality level and from the preventive activities to the solution of the crisis situation). In the context of the climate change and the projected increase of the number of the future disasters, emphasising the responsibility of individuals for self-protection, promoting interest in their own resilience, and emphasising the role of a responsible individual within society appear to be inevitable. After supporting individual’s position in the DRR system, it is, subsequently, possible to enforce also all-society-engaging initiatives. However, without the implementation of the first mentioned point (raising the awareness of the society), it will be a difficult task, and therefore, it is appropriate to carry out these activities simultaneously or in close connection.

The results of the present study suggest that especially bad experience trigger a protective behaviour for the future disasters. The major damages on the property caused by the floods and, in a smaller extent, the threats of life and health are the predominant reasons for an active approach to strengthening the safety and one’s own resilience. However, discrepancies between the subjective and objective perception of the seriousness of the disaster experienced can be observed. The subjective assessment of the seriousness of the consequences experienced seems to be overestimated from the point of view of the major floods’ statistics in the SR (there was only a few major floods [13]). The subjective perception of impacts can be, therefore, considered as a more important factor considered than only statistical data from previous events. In this regard, the official statistics should be reconsidered for the purposes of disaster preparedness assessment; and subjective perspective should not be overlooked by the policy makers in the future proposals of building the disaster resilience.

Last but not least, the specifics of the vulnerable population groups must be considered in the development of disaster risk management initiatives, e.g., the study confirms low protective behaviour in association with economically vulnerable group of the population. It was not confirmed for the families with disabled people in household or with a need of increased care, however, vulnerable groups of the population can be identified also from other perspectives (e.g., present study does not include older people; does not assess completeness of the family, etc.). Adequately, strengthening the preparedness and resilience of the society, therefore, requires a deeper assessment of the vulnerable population groups in the given country and the adjustment of the state support for these groups of concern.

In summary, only a few of the aforementioned requirements will be possible to address if the legal environment of the DRR issue (in the SR) remains unchanged. The change of the duties, competencies, and especially resource allocation (financial and personnel) is required. In the conditions of the SR, the strengthening of the position of the municipalities is the most desirable in order to ensure close cooperation with the population and its engagement into the DRR activities in the given region.

### 5.2. Limitations of the Study

This study is limited by the findings that concern the fact if the respondents took any protective measures or not. We did not investigate the extent of these measures and if they were sufficient or not. However, the primary goal was to deduce the willingness (effort) of people to participate in adopting these measures individually. The probability of the community solidarity and participation in building a disaster-resilient society results from this fact. At the same time, the study aimed at investigating the reasons for the proactive behaviour and protection motivation from the long-term point of view (long-term hazard adjustment) rather than an immediate disaster response that could affect certain respondents whose attitudes to the risk and risk perception could be (due to the time gap since the negative experience) partially distorted. At the same time, the findings of the study are limited on the “youth and adults” population as we assume that these groups have the greatest potential (physical, societal, or economical) to be an effective part of enhancing disaster preparedness of the whole population in the future. In addition, the differences between these two groups were investigated. The study was focused on the population perspective only; the public authorities’ influence was not investigated.

## 6. Conclusions

In total, the identified linkages and facts should be a driving engine of the internal motivation of people, but at the same time, also an opportunity of the competent authorities to face the problem directly and fight the conceptual and strategic shortages and problems that have stacked in the given area recently. From the governmental point of view the basic problem is the total refusal of the preparation system being in existence since 1989 and the subsequent inactivity of the responsible bodies in this area for a long time period. There is no complex conception of preparation and education of the population for disasters and this study shows the consequences caused by this fact on the basis of the aforementioned questionnaire research. After the adoption of systemic changes at the government level, it is possible to approach solutions at the local level. The current setting of the DRR system in the SR does not allow to start from the bottom-to-up change, as the competencies and resources of the local authorities are very limited. The findings reveal the causal factors affecting the actual preparedness state and the causal factors that influence the proactive behaviour of the people against the risk. The regression analysis reveals that the factors influencing the preventive behaviour are age, social status, economic situation, the worst experience, all disaster awareness variables, fears of occurring disasters in the future, and subjective assessment of own preparedness for disasters. The findings also uncover the low level of the information rate about disasters and knowledge about the probable threats and recommended possibilities of protection. In terms of the risk perception, people feel relatively safe and do not assume any significant threats in the nearest future. However, they are not sure about the impacts of the climate change in the SR. These impacts are for them more transparent from the global perspective. People do not admit that also in the SR, the impacts of these changes can be more serious and should prepare for them better. The things that drive their active attitude to preparing for disasters is first of all their experience and especially the experience of a more serious character, e.g., major damages on the property and threat of life and health. Although not all the acquired results and findings are consistent with other studies, several current statements have been confirmed and this fact can serve for improving the knowledge basis regarding to the area of population disaster preparedness.

The research results emphasise that the strengthening of the preparedness is affected by the economic situation of the respondents, the experience with disasters, and the population’s awareness level about disasters. The addressing of the population awareness is considered the basic point towards the future development of DRR system. For us to increase this awareness, it is inevitable to emphasise the role of the individual person and the whole society in the system. The educational campaigns should emphasise the personal responsibility of the individuals for self-protection and, at the same time, their important role within the society. For people, it is important to receive relevant information about the protection possibilities, particularly in the context of the climate change. The possibilities of protection should be offered with regard to the individual possibilities of people because, as the research showed, the realisation of some measures could be a problem for the more economically vulnerable groups. The outputs of the people’s engagement within the society for achieving the common goal should be emphasised.

Although the population preparedness is generally rather weak nowadays, several predictors of this state can be addressed by the new or existing conceptions and appropriately selected ways and methods that will be systematically incorporated to the population’s preparation. The findings require interventions on behalf of the competent bodies or the population itself. This is the way how the population’s resilience could be improved and can help mitigate the potential impacts and protect the most precious human values and interests in the future.

The area of protecting and preparing the society is an interdisciplinary problem because the linkages between the crisis management system and the educational system are at least visible. Therefore, the further interest of the investigators is to inquire into the suitable forms of education in the field of disaster protection of various groups of the population.

Another aim of the authors is to investigate the differences of the adaptive behaviour that are motivated by various types of disasters or extreme weather events and their particular properties (e.g., the speed of the start, the impact extent, duration of the impact, expected personal impacts, etc.) that are significantly emphasised by Lindell and Perry (PADM model) [117]. There are certain transparent differences of the results; however, the confirmation of these indications requires a deeper analysis.

## Figures and Tables

**Table 1:** Questionnaire structure concerning the preparedness aspects.

Preparedness Aspect	Main Variables	Response
Experience	Experience with disasters	Frequency
	The worst experienced disaster impacts	None (1), logistic complications (transport, services, etc.), minor damages on property, major damages on property (including damaged environment), and threats for life and health (5)
	Evacuation experience	Yes/no
Disaster awareness	Flood maps awareness	Yes/no/do not know
	Evacuation plan awareness	Yes/no/do not know
	Hazard-prone areas awareness	Yes/no/do not know
	Knowledge of warning signals	Assigning correct answer in the table
	Protection possibilities awareness	Yes/no
Risk perception	Fears of occurring disasters in the future	Scale from 1 to 5 *
	Probability of occurring disasters during next 3–5 years	Scale from 1 to 5 *
	Occurrence of negative events connected with climate change	Scale from 1 to 5 *
	Subjective assessment of own preparedness for disasters	Scale from 1 to 5 *
	Feeling of safety	Scale from 1 to 5 *
Proactive behaviour	Adopted preventive and mitigation measures	Yes (description of particular activity)/no

* 5 is higher fear, probability, occurrence, subjective assessment, safety.

**Table 2:** Basic attributes and characteristics of the respondents.

Basic Characteristics	*n* = 794	Percentage
Gender	Male	52.39%
	Female	47.61%
Age	Mean + SD	29.35 + 11.36
	From 18 to 24	52.14%
	More than 24	47.86%
Education	Basic	0.38%
	Secondary school	59.82%
	University	39.80%
Social status	Adults	54.65%
	Youth (students)	45.34%
Location of living	Urban	51.64%
	Rural	48.36%
Type of living	House	57.18%
	Flat	42.82%
Disabled people or persons with increased need of care	Yes	39.29%
	No	60.71%
	Exact number in the household	Mean (mode), SD 0.63 (0), 0.96
Economic situation	1	6.42%
	2	33.63%
	3	31.61%
	4	22.04%
	5 (5 is better)	6.30%

**Table 3:** Main and additional variables concerning the risk perception.

**Main Variables**	**Mean**	**SD**
Fears of disasters in the future	2.25	0.81
Probability of occurring disasters during next 3–5 years	3.00	0.64
Occurrence of negative events in connection with climate change	3.31	0.89
Subjective assessment of the respondent’s preparedness for disasters	2.60	1.01
Feeling of safety	4.08	0.88
**Additional Variables**	**Mean**	**SD**
Rate of demonstration of climate change in the SR	3.45	0.94
Rate of demonstration of climate change in Europe	3.89	0.84
Rate of demonstration of climate change worldwide	4.35	0.89

**Table 4:** Association and correlation between the dependent variable and basic attributes and characteristics of the respondents.

Basic Attributes and Characteristics of the Respondents									
C.	Gender	Age	Education	Social Status	Location of Living	Living Place	Number of People in Household	Number of Disabled People or People with Need of Increased Care in Household	Economic Situation
*V*	0.00	*r_s_* = −0.34 **	0.01 *	0.23 **	0.01 *	0.01 *	*r_s_ =* 0.15 **	*r_s_* = −0.02	*r_s_ =* 0.21 **
*r*	−0.04	−0.26 **	−0.10 **	−0.48 **	0.10 **	0.08 *	0.13 **	−0.03	0.27 **

** *p* < 0.01; * *p* < 0.05.

**Table 5:** Multiple logistic regression analysis investigating factors (basic attributes and characteristics of the respondents) relating to respondent’s preventive behaviour and disaster preparedness.

Variables	B	S.E.	*p*	Exp.(B)	95% C.I. for Exp(B)	
					Lower	Upper
Age	0.025	0.011	0.026 *	1.026	1.003	1.049
Gender	−0.263	0.176	0.134	0.769	0.545	1.084
Education	0.004	0.189	0.982	1.004	0.693	1.455
Social status	−2.543	0.268	0.000 **	0.079	0.046	0.133
Location of living	−0.096	0.252	0.703	0.908	0.554	1.488
Living place	0.106	0.261	0.685	1.111	0.667	1.853
Number of people in household	0.062	0.073	0.396	1.064	0.922	1.228
Number of disabled people in household (or with a need of increased care)	0.083	0.101	0.411	1.087	0.891	1.325
Economic situation	0.368	0.092	0.000 **	1.445	1.206	1.730
Constant	−1.385	0.481	0.004	0.250		

B—regression coefficient; S.E.—standard error; C.I.—confidence interval; ** *p* < 0.01; * *p* < 0.05.

**Table 6:** Association and correlation between dependent variable and disaster experience variables.

Disaster Experience Variables			
C.	Experience Number	The Worst Experience	Evacuation Experience
*V*	r_s_ = 0.14 **	0.47 **	0.07
*r*	0.12 **	0.44 **	0.07

** *p* < 0.01.

**Table 7:** Multiple logistic regression analysis investigating factors (disaster experience variables) relating to respondent’s preventive behaviour and disaster preparedness.

Variables	B	S.E.	*p*	Exp.(B)	95% C.I. for Exp(B)	
					Lower	Upper
Experience number	−0.122	0.078	0.121	0.856	0.759	1.033
The worst experience	0.741	0.066	0.000 **	2.099	1.844	2.388
Evacuation experience	0.174	0.291	0.551	1.189	0.673	2.103
Constant	−2.539	0.197	0.000			

B—regression coefficient; S.E.—standard error; C.I.—confidence interval; ** *p* < 0.01.

**Table 8:** Association and correlation between the dependent variable and disaster awareness variables.

Disaster Awareness					
C.	Flood Maps Awareness	Evacuation Plan Awareness	Hazard-Prone Areas Awareness	Warning Signals Awareness	Protection Possibilities Awareness
*V*	0.07 **	0.07 **	0.11 **	0.03 **	0.02 **
*r*	0.27 **	0.27 **	0.19 **	0.17 **	−0.14 **

** *p* < 0.01; * *p* < 0.05.

**Table 9:** Multiple logistic regression analysis investigating factors (disaster awareness variables) relating to respondent’s preventive behaviour and disaster preparedness.

Variables in the Equation	B	S.E.	*p*	Exp.(B)	95% C.I. for Exp(B)	
					Lower	Upper
Hazard-prone areas awareness	0.607	0.193	0.002 **	1.835	1.258	2.677
Flood maps awareness	0.789	0.196	0.000 **	2.201	1.498	3.234
Evacuation plan awareness	0.698	0.143	0.000 **	2.009	1.519	2.658
Protection possibilities awareness	−1.444	0.260	0.000 **	0.236	0.142	0.393
Warning signals awareness	0.569	0.192	0.003 **	1.767	1.213	2.573
Constant	−1.337	0.121	0.000 **	0.263		

B—regression coefficient; S.E.—standard error; C.I.—confidence interval; ** *p* < 0.01.

**Table 10:** Association and correlation between the dependent variable and risk perception variables.

Disaster Perception							
C.	Fears of Occurring Disasters in the Future	Probability of Occurring Disasters during Next 3–5 Years	Subjective Assessment of Own Preparedness for Disasters	Feeling of Safety	Rate of Demonstration of Climate Change		
					in the SR	in the EU	World-Wide
*r_s_*	0.14 **	0.05	0.29 **	−0.04	0.08 *	0.05	0.03
*r*	0.11 **	0.05	0.28 **	−0.02	0.08 *	0.07	0.06

** *p* < 0.01; * *p* < 0.05.

**Table 11:** Multiple logistic regression analysis investigating factors (disaster perception variables) relating to respondent’s preventive behaviour and disaster preparedness.

Variables in the Equation	B	S.E.	*p*	Exp.(B)	95% C.I. for Exp(B)	
					Lower	Upper
Fears of occurring disasters in the future	0.301	0.085	0.000 **	1.351	1.144	1.597
Probability of occurring disasters during next 3–5 years	−0.020	0.088	0.824	0.981	0.825	1.166
Subjective assessment of own preparedness for disasters	0.692	0.087	0.000 **	1.998	1.685	2.368
Feeling of safety	−0.018	0.095	0.849	0.982	0.815	1.183
Rate of demonstration of climate change in the SR	0.165	0.089	0.065	1.179	0.990	1.404
Constant	−3.677	0.0656	0.000	0.025		

B—regression coefficient; S.E.—standard error; C.I.—confidence interval; ** *p* < 0.01.

## Data Availability

The data presented in this study are available on request from the corresponding author.
